# Supplementary material for: A Sarcoptes scabiei specific isothermal amplification assay for detection of this important ectoparasite of wombats and other animals
Source: PeerJ. 2018 Jul 27;6:e5291. doi: 10.7717/peerj.5291 (PMC6065476; doi:10.7717/peerj.5291)
Supplement: Table S1 [file peerj-06-5291-s001.docx]

| Haplotype number | Number of sequences | Genbank Accession number |
| --- | --- | --- |
| 1 | 1 | AM980728 |
| 2 | 6 | AB820967, AM980803, AM980806, AM980812, AF129151, KJ739610 |
| 3 | 3 | AF387705, AB535737, AF387719 |
| 4 | 1 | AM980775 |
| 5 | 1 | AM980818 |
| 6 | 35 | AB778900, AB778901, AB778898, AB778899, AF387723, AF387722, AF387724, AF387725, AF387703, AM980686, AF129145, AF129149, AF129148, AF129150, AF129147, AF129142, AF129143, AM980731, AB820972, AB778917, AF129164, AF129163, AF129152, AF387712, AF129160, AF129154, AF129153, AB778896, AB778897, AB778895, AB778905, AB778912, AF387704, AF129157, AM980750 |
| 7 | 2 | AF129161, KJ739612 |
| 8 | 1 | AM980679 |
| 9 | 1 | AF129146 |
| 10 | 4 | AM980689, AF129156, AF129155, AF129158 |
| 11 | 1 | AM980758 |
| 12 | 1 | AM980799 |
| 13 | 21 | AB778902, AB778903, AF387730, AB778904, AB778906, AB778907, AB778908, AB778909, AB778910, AB778911, AB778913, AB778914, AB778915, AM980757, AB778916, AB778918, AM980681, AB778919, AF129159, AF387729, AF387727 |
| 14 | 1 | AM980808 |
| 15 | 1 | AM980773 |
| 16 | 2 | AM980721, AM980751 |
| 17 | 1 | AM980770 |
| 18 | 1 | AF129144 |
| 19 | 1 | KJ739613 |
| 20 | 1 | KJ739611 |
| 21 | 1 | KJ739615 |
