# Supplementary material for: A Sarcoptes scabiei specific isothermal amplification assay for detection of this important ectoparasite of wombats and other animals
Source: PeerJ. 2018 Jul 27;6:e5291. doi: 10.7717/peerj.5291 (PMC6065476; doi:10.7717/peerj.5291)
Supplement: Table S2 — Each sample has a corresponding host, microscopy mite count, skin scraping PCR and LAMP result. In some instances, multiple skin scrapings were taken from an individual, reflected by the sample name_body location. LF: left flank, RF: right flank, N: neck, H: head, RA: right forearm, RL: right hind leg, B: back, LL: left hind leg. If multiple skin scrapings were taken from the same body location this is described by the number next to name_body location. [file peerj-06-5291-s002.docx]

**Results for microscopy mite count, PCR and LAMP for all samples used in this study.** Each sample has a corresponding host, microscopy mite count, skin scraping PCR and LAMP result. In some instances, multiple skin scrapings were taken from an individual, reflected by the sample name_body location. LF: left flank, RF: right flank, N: neck, H: head, RA: right forearm, RL: right hind leg, B: back, LL: left hind leg. If multiple skin scrapings were taken from the same body location this is described by the number next to name_body location.

| **Host** | **Sample Name** | **Mite Count** | **Skin Scraping 374PCR** | **LAMP** | **Time to amplify (min)** | **Melt (°C)** |
| --- | --- | --- | --- | --- | --- | --- |
| Wombat | DW02_RL1 | 11 | Positive | Positive | 10.15 | 85.34 |
| Wombat | DW02_RL2 | NA | Positive | Positive | 11.15 | 85.54 |
| Wombat | W002_LF | 0 | Negative | Negative |  |  |
| Wombat | W002_RF | 2 | Positive | Positive | 15.30 | 85.51 |
| Wombat | W003_RF | 13 | Positive | Positive | 11.00 | 85.65 |
| Wombat | W004_N | 2 | Positive | Positive | 12.30 | 85.32 |
| Wombat | W004_RF | 0 | Negative | Positive | 14.30 | 85.36 |
| Wombat | W005_RF | 13 | Positive | Positive | 11.45 | 85.47 |
| Wombat | W006_H | 0 | Positive | Positive | 20.30 | 85.43 |
| Wombat | W006_RF | 2 | Positive | Positive | 11.45 | 85.48 |
| Wombat | W008_RF | 8 | Positive | Positive | 12.00 | 85.43 |
| Wombat | W009_RF | 0 | Negative | Negative |  |  |
| Wombat | W010_RF | 0 | Negative | Negative |  |  |
| Wombat | W013_LF | 0 | Negative | Negative |  |  |
| Wombat | W013_RA | 0 | Negative | Negative |  |  |
| Wombat | W014_LF | 0 | Negative | Negative |  |  |
| Wombat | W014_RL | 0 | Negative | Negative |  |  |
| Wombat | W015_RA | 0 | Positive | Positive | 13.15 | 85.18 |
| Wombat | W016_LF | 1 | Positive | Positive | 11.45 | 85.45 |
| Wombat | W016_N | 7 | Positive | Positive | 11.00 | 85.33 |
| Wombat | W016_RL | 4 | Positive | Positive | 13.00 | 85.20 |
| Wombat | W017_B | 0 | Negative | Positive | No time recorded | 85.78 |
| Wombat | W017_RL | 0 | Negative | Negative |  |  |
| Wombat | W018_RF | 0 | Negative | Negative |  |  |
| Wombat | W019_RA | 0 | Negative | Negative |  |  |
| Wombat | W019_RF | 0 | Negative | Negative |  |  |
| Wombat | W020_RF | 0 | Negative | Negative |  |  |
| Wombat | W021_RL | 11 | Positive | Positive | 12.00 | 85.28 |
| Wombat | W021_RF | 7 | Positive | Positive | 10.45 | 85.48 |
| Wombat | W022_LL | 0 | Negative | Negative |  |  |
| Wombat | W023_LL1 | 0 | Negative | Negative |  |  |
| Wombat | W023_LL2 | 0 | Negative | Negative |  |  |
| Wombat | W023_LL3 | 0 | Negative | Negative |  |  |
| Wombat | W024_RF1 | 0 | Negative | Negative |  |  |
| Wombat | W024_RF2 | 0 | Positive | Positive | 15.00 | 85.80 |
| Wombat | W025_LF1 | 0 | Negative | Negative |  |  |
| Wombat | W025_LF2 | 0 | Negative | Negative |  |  |
| Wombat | W025_LF3 | 0 | Negative | Negative |  |  |
| Wombat | W027_RF1 | 3 | Positive | Positive | 12.30 | 85.29 |
| Wombat | W027_RF2 | 1 | Positive | Positive | 14.00 | 85.33 |
| Wombat | W027_RF3 | 0 | Positive | Positive | 20.00 | 85.08 |
| Dog | NT1 | NA | Positive | Positive | 12.00 | 85.42 |
| Dog | NT2 | NA | Positive | Positive | 15.00 | 85.62 |
| Dog | NT3 | NA | Negative | Negative |  |  |
| Dog | NT4 | NA | Positive | Positive | No time recorded | 85.54 |
| Dog | NT5 | NA | Positive | Positive | 14.45 | 85.68 |
| Koala | KSA1 | NA | Positive | Positive | 11.00 | 85.40 |
| Wombat | WV1 | NA | Negative | Negative |  |  |
| Wombat | WV2 | NA | Negative | Negative |  |  |
| Wombat | WV3 | NA | Negative | Positive | No time recorded | 85.08 |
| Wombat | WV4 | NA | Negative | Negative |  |  |
| Wombat | WV5 | NA | Negative | Negative |  |  |
| Wombat | WV6 | NA | Negative | Negative |  |  |
| Wombat | WV7 | NA | Negative | Negative |  |  |
| Wallaby | WaV1 | NA | Negative | Negative |  |  |
| Wallaby | WaV2 | NA | Negative | Negative |  |  |
| Koala | KV1 | NA | Negative | Negative |  |  |
| Wombat (negative) | WT1 | 0 | Negative | Negative |  |  |
| Wombat (negative) | WT2 | 0 | Negative | Negative |  |  |
| Wombat (negative) | WT3 | 0 | Negative | Negative |  |  |
| Wombat (negative) | WT4 | 0 | Negative | Negative |  |  |
| Wombat (negative) | WT5 | 0 | Negative | Negative |  |  |
| Wombat (negative) | WT6 | 0 | Negative | Negative |  |  |
| Wombat (negative) | WT7 | 0 | Negative | Negative |  |  |
| *S. scabiei* | Ss2 | 1 | Positive | Positive | 20.00 | 85.26 |
| *S. scabiei* | Ss3 | 3 | Positive | Positive | 11.30 | 85.39 |
