# Supplementary material for: A Sarcoptes scabiei specific isothermal amplification assay for detection of this important ectoparasite of wombats and other animals
Source: PeerJ. 2018 Jul 27;6:e5291. doi: 10.7717/peerj.5291 (PMC6065476; doi:10.7717/peerj.5291)
Supplement: Table S3 [file peerj-06-5291-s003.docx]

| Sample | Time to amplify (min) | Melt (°C) |
| --- | --- | --- |
| W019_RF | 12:30 | 85.42 |
| WT3 | 13:45 | 85.57 |
| WV4 | 11:15 | 85.52 |
| NT3 | 11:15 | 85.67 |
| WaV1 | 16:15 | 85.28 |
| W017_RL | 11:15 | 85.33 |

**LAMP results of “spiked” negative samples to eliminate assay inhibition.**
