# Supplementary material for: A Sarcoptes scabiei specific isothermal amplification assay for detection of this important ectoparasite of wombats and other animals
Source: PeerJ. 2018 Jul 27;6:e5291. doi: 10.7717/peerj.5291 (PMC6065476; doi:10.7717/peerj.5291)
Supplement: Table S4 [file peerj-06-5291-s004.docx]

**Reproducibility of *S. scabiei* LAMP using skin scraping DNA extracts and mite only DNA.**

| Samples | Run | Time to amplify (min) | Melt (°C) | Time (Mean + SD) | Melt (Mean + SD) |
| --- | --- | --- | --- | --- | --- |
| SS2 | 1 | 21.15 | 85.03 | 20.48, 0.60 | 85.19, 0.14 |
|  | 2 | 20 | 85.26 |  |  |
|  | 3 | 20.3 | 85.27 |  |  |
| W024_RF2 | 1 | 15 | 85.3 | 13.38, 1.51 | 85.19, 0.27 |
|  | 2 | 12 | 84.88 |  |  |
|  | 3 | 13.15 | 85.38 |  |  |
| NT3 | 1 | Negative |  |  |  |
|  | 2 | Negative |  |  |  |
|  | 3 | Negative |  |  |  |
| W002_RF | 1 | 15.15 | 85.48 | 15.15, 0.15 | 85.38, 0.10 |
|  | 2 | 15 | 85.28 |  |  |
|  | 3 | 15.3 | 85.37 |  |  |
| W002_LF | 1 | Negative |  |  |  |
|  | 2 | Negative |  |  |  |
|  | 3 | Negative |  |  |  |
| W021_RL | 1 | 9.15 | 85.14 | 9.15, 0 | 85.22, 0.18 |
|  | 2 | 9.15 | 85.09 |  |  |
|  | 3 | 9.15 | 85.42 |  |  |
| W023_LL1 | 1 | Negative |  |  |  |
|  | 2 | Negative |  |  |  |
|  | 3 | Negative |  |  |  |
| WT6 | 1 | Negative |  |  |  |
|  | 2 | Negative |  |  |  |
|  | 3 | Negative |  |  |  |
| WV3 | 1 | No time recorded | 84.78 | No time recorded | 85.00, 0.22 |
|  | 2 | No time recorded | 85.01 |  |  |
|  | 3 | No time recorded | 85.22 |  |  |
| WV4 | 1 | Negative |  |  |  |
|  | 2 | Negative |  |  |  |
|  | 3 | Negative |  |  |  |
| KSA1 | 1 | 11 | 85.4 | 11.43, 0.51 | 85.40, 0.02 |
|  | 2 | 12 | 85.38 |  |  |
|  | 3 | 11.3 | 85.41 |  |  |
| W014_LF | 1 | Negative |  |  |  |
|  | 2 | Negative |  |  |  |
|  | 3 | Negative |  |  |  |
| W025_LF2 | 1 | Negative |  |  |  |
|  | 2 | Negative |  |  |  |
|  | 3 | Negative |  |  |  |
| W027_RF2 | 1 | 14 | 85.33 | 12.20, 1.57 | 85.24, 0.08 |
|  | 2 | 11.15 | 85.23 |  |  |
|  | 3 | 11.45 | 85.17 |  |  |
