# Supplementary figures and images for: A Sarcoptes scabiei specific isothermal amplification assay for detection of this important ectoparasite of wombats and other animals
Source: PeerJ. 2018 Jul 27;6:e5291. doi: 10.7717/peerj.5291 (PMC6065476; doi:10.7717/peerj.5291)

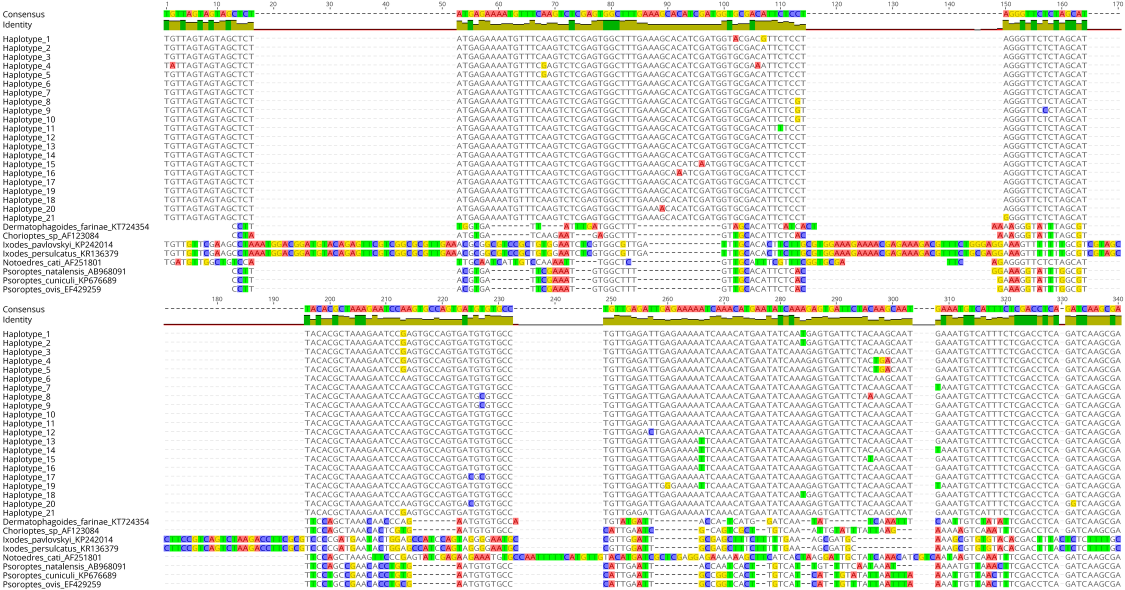

Supplement: Figure S1 [file peerj-06-5291-s005.png]

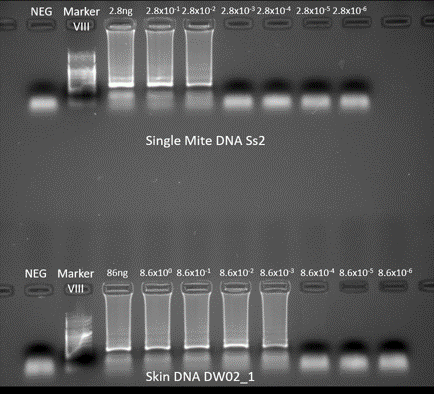

Supplement: Figure S2 — UV visualisation of a single mite (Ss2: 2.8 ng/µL) and a S. scabiei positive wombat skin scraping (DW02_1: 86 ng/µL) LAMP amplicons on an ethidium bromide stained agarose gel. DNA serial dilution LAMP assays were performed on a thermal block for 30 minutes at 65 °C. Two negative controls containing water were also included. DNA Molecular Weight Marker VIII (Sigma-Aldrich) was used. [file peerj-06-5291-s006.png]
